# Social connection before and after the COVID-19 pandemic: results of the Belgian Health Interview Survey

**DOI:** 10.1186/s12889-026-26932-1

**Published:** 2026-03-18

**Authors:** Kirsten A. Verhaegen, Maaike Paredis, Valentien Taeldeman, Stefaan Demarest, Rana Charafeddine, Piet Bracke, Katrijn Delaruelle, Elise Braekman

**Affiliations:** 1 Department of Epidemiology and Public Health, Sciensano, Juliette Wytsmanstraat 14, Brussels, 1050 Belgium; 2 Department of Sociology, Faculty of Political and Social Sciences, UGent, Sint-Pietersnieuwstraat 41, Ghent, 9000 Belgium

**Keywords:** Social connection, COVID-19 pandemic, Health survey, Social contact frequency, Social satisfaction, Social support, Post-pandemic, Youth, Living alone

## Abstract

**Background:**

The COVID-19 pandemic required major preventive measures that had a substantial impact on social life. Since then, there has been concern for pandemic side-effects on social connection. While short-term increases in poor social connection have been observed during the pandemic, less is known about long-term consequences. Only a few studies have made comparisons of social connection before, during and after the pandemic, showing conflicting results. Some observed lasting decreases in social connection, while others observed recovery.

**Methods:**

The current study aimed to assess changes in social connection, by comparing pre- and post-pandemic levels of social contact frequency, social support (measured with the Oslo Social Support Scale) and social satisfaction. Logistic regression was performed using cross-sectional data from the Belgian Health Interview Surveys, with a pre-pandemic measurement in 2018 (*N* = 8075) and a post-pandemic measurement in 2023–2024 (*N* = 4926) in the general Belgian population aged 15 years or older, and in separate sociodemographic subgroups.

**Results:**

In the general population, there were no differences in social connection between the pre- and post-pandemic waves. However, stratified analyses showed lower post-pandemic social satisfaction in those with tertiary education and those living alone as compared to pre-pandemic levels. In addition, in 15 to 24 year-olds, the odds of poor social support were higher after the pandemic, as compared to before. Our findings argue for particular attention for the social connection of adolescents and young adults, and those living alone, both in the aftermath of the COVID-19 pandemic, and in anticipation of future pandemics. Additional research is needed to understand the post-pandemic drop in social satisfaction prevalence in those with tertiary education.

**Conclusions:**

Taken together, this study contributes to longer-term insights on social connection in the context of COVID-19 pandemic, preparedness for future pandemics and particular points of concern for social connection in general.

**Supplementary Information:**

The online version contains supplementary material available at 10.1186/s12889-026-26932-1.

## Background

As the COVID-19 pandemic unfolded, the spread of the SARS-CoV-2 virus urged many countries to adopt preventive measures on a wide and radical scale, ranging from physical distancing and face mask wearing to school, workplace and shop closures and lockdowns [1–3]. As many of these measures targeted social habits (e.g., reducing number of social contacts, physical distancing), there has been particular attention for the consequences of decreased social connection as a potential side-effect of these measures, with calls for awareness in the media [4–6] and in research [7, 8]. These concerns about social connection during and after the pandemic were rooted in the knowledge that poor social connection has been found to strongly predict physical and mental health [9–11].

To fully understand the repercussions of the pandemic on social connection, we need to acknowledge its multidimensional nature. In fact, social connection is an umbrella term for indicators that describe one’s social life [12] and has been studied using various indicators. According to the framework proposed by Holt-Lunstad [11], social connection has a structural, functional and qualitative component. Due to data limitations, the current study is focused on the structural and functional components. The structural component describes an individual’s social life in terms of properties such as social network size and diversity, housing situation, relationship status and frequency of social contacts. Poor structural connection is indicated by social isolation, which refers to having a social network that is small or infrequently engaged with [13], or a low frequency of social contacts. The functional component refers to the extent to which the social network provides help and resources, and meets several needs of the individual (i.e., what functions it serves). This is often studied using loneliness, received or perceived social support and social satisfaction (i.e., overall satisfaction with the contacts in the social network). The qualitative component describes the positive and negative circumstances that determine relational quality in specific interactions, such as relationship satisfaction or distress in a romantic relationship, social exclusion in a group, or discrimination in public life. When studying social connection, it is important to clearly distinguish these components, and identify the studied component at hand. After all, their indicators are often only moderately correlated [14, 15]. For example, someone with frequent social contacts or a large social network can still feel lonely, or someone with a large and supportive social network might still be affected by social exclusion in a specific social group, such as the workplace. While a question on people’s satisfaction with social life in general can serve as an indicator of the functional component, a question on how people perceive their partner relationship would serve as an indicator of the qualitative component. Unfortunately, we cannot measure the latter with the data at hand.

Since the pandemic, several studies have demonstrated short-term consequences of the COVID-19 pandemic, focusing on these different components of social connection. First, studies on structural social connection showed drastic reductions in mobility and social activity after the enforcement of lockdown policies [16–18]. This resulted in higher levels of social isolation in comparison to pre-pandemic levels [19, 20]. Second, functional social connection was also negatively impacted. Systematic reviews repeatedly showed that levels of loneliness were increased during the pandemic [21, 22]. Among the few studies on the evolution of social support during the pandemic, some have found decreased social support during the COVID-19 pandemic, although others found no such change [23, 24]. Third, qualitative social connection also seems to have been under pressure. For example, there have been reports of increased domestic violence [25, 26] and relationship stress [27].

However, concerns were raised not only over the short-term, but also over the long-term consequences of COVID-19 preventive measures for social connection. The aforementioned studies compared levels during the pandemic with levels before the pandemic, but to assess the long-term impact, a comparison on a longer timescale is needed. Such studies including post-pandemic social connection are only starting to emerge. Structural social connection seems to have recovered only partly. An international study conducted in 2022 in the United Kingdom, Belgium and The Netherlands, showed that the average number of social contacts had not yet recovered to pre-pandemic levels [28]. In another study in The Netherlands, the number of social contacts had not recovered to pre-pandemic levels by 2023 [29]. This was particularly true among adults aged 20 to 59, potentially due to an increase in teleworking since the pandemic. The few findings on functional social connection are still inconclusive and mostly focused on loneliness. In Slovenia, pandemic increases in social isolation and loneliness had not dropped to pre-pandemic levels in 2023 [30]. However, in The Netherlands, pandemic increases in loneliness did return to pre-pandemic levels [31]. Studies on long-term changes (i.e., including a post-pandemic measurement) in qualitative social connection have yet to emerge.

As the current literature is still inconclusive and no comprehensive Belgian studies exist yet, we studied the evolution of social connection in Belgium in the peri-pandemic period (i.e., comparing pre- and post-pandemic levels). To do so, we used data from the Belgian Health Interview Surveys (BHIS), a cross-sectional health survey in the general Belgian population. Since the social connection module of the BHIS only contains information on structural and functional social connection, the current study focuses on these components. Based on existing findings about social activity patterns [28, 29], we hypothesized decreased post-pandemic levels of structural social connection compared to pre-pandemic levels, particularly in the working adult population (H1). More specifically, we expected a higher prevalence of infrequent social contacts in the post-pandemic wave compared to the pre-pandemic wave. Additionally, we tentatively expected to find similar pre- and post-pandemic levels of functional social connection in the general Belgian population (H2). More specifically, we expected a similar prevalence of poor perceived social support and social satisfaction.

Another element of social connection concerns was the vulnerability of specific sociodemographic subgroups in society. First, those living alone might be at risk, because of their dependency on social contacts outside the household for meeting one’s social needs. Indeed, living alone has been found to be a risk factor for loneliness, both before and during the COVID-19 pandemic [32–34]. Second, youth could be more vulnerable for poor social connection after the COVID-19 pandemic. Adolescents might be more at risk of poor functional social connection because close social contacts are a crucial element of their developmental stage [35]. Since adolescent loneliness and feelings of social isolation have been seen to be increased during the COVID-19 pandemic [36, 37], there is concern that this may have hindered social development [38]. Although many adolescents and young adults may have returned to more in-person forms of education or work, this social life disturbance during a developmentally crucial period in their lives might have caused longer-term functional social connection problems [38]. Similarly, young-adult students might also be more at risk because of the highly social nature of student life [39, 40]. Several studies have been confirmed this [32, 41–43]. Third, concerns were also raised over the structural and functional social connection of older adults [44]. Indeed, both structural and functional social connection were affected in older adults during the COVID-19 pandemic [45]. However, it is still debated whether older age is a direct risk factor for loneliness in general [33]. Fourth, in men and women, social connection problems might manifest differently. More specifically, being male has been found to be a risk factor for poor structural social connection, while being female seems to be a risk factor for poor functional social connection, such as (emotional) loneliness [46–49]. In the context of the COVID-19 pandemic, being female has been identified as a risk factor for increased feelings of loneliness [32]. Fifth, having a lower degree of education has been found to be a general risk factor for poor structural and functional social connection [49, 50]. The literature on pandemic social inequalities showed that those with lower education (among other groups) were more vulnerable in terms of mortality [51, 52] and economic impact [53]. Similarly, it is possible that this is also the case specifically for impacts on structural and functional social connection. In the limited literature on this topic, some have indeed observed such differences (e.g., [32]).

However, as is the case with research on overall time trends since the COVID-19 pandemic, longer-term trends have not been assessed yet. For this reason, we additionally assessed whether decreases in structural and functional social connection since the pre-pandemic period can be observed for specific groups in terms of household situation, age, sex and education. Based on previous research on risk factors for (pandemic) social isolation and loneliness (e.g., [32]), we determined those living alone, those between 15 and 24 years old (i.e., in late adolescence to young adulthood), older adults (i.e., those aged 65 years and older), women and those without tertiary education as target subgroups for which we expect lower levels of social connection after the pandemic, compared to before (H3), for which the main analyses were repeated in a stratified manner.

## Methods

### Survey design

The current study made use of data from the BHIS, a national household survey conducted every five years in Belgium. Since 1997, it is organized by Sciensano, the Belgian Institute of Public Health, and commissioned by the Belgian federal and regional governments. In this study, the 2018 wave was used as a pre-pandemic time point, whereas the 2023–2024 wave was used as the post-pandemic time point. The surveys consist of several modules on physical, mental and social health, as well as on health care consumption and health determinants. Only the variables used in the current study are discussed here. More methodological details of the BHIS can be found in Demarest et al. [54, 55].

### Participants

The 2018 wave contained 8075 participants between the age of 15 and 101 (*M* = 50.78, *SD* = 18.51), among which 53% women (for further sociodemographic description of the sample, see Table 1 in Supplementary Material (SM) 1). The 2023–2024 wave contained 4926 participants between the age of 15 and 100 (*M* = 52.81, *SD* = 18.80), with 53% women. All participants were Belgian residents, sampled from the national registry, and completed the survey in Dutch, French or German. Participants were recruited from the national register using multi-stage, stratified sampling. They read and agreed with the informed consent prior to participation, in accordance with the Declaration of Helsinki [56]. The BHIS data collection uses a combination of face-to-face interviews and self-administered paper-and-pencil questionnaires for more sensitive questions. All social connection questions were self-administered, whereas the sociodemographic variables were collected face-to-face. The dataset was weighted based on age, sex, household size, region, timing of the interview, education level.

### Social connection

Structural social connection was measured with a question on social frequency (see SM 2). From this, a binary social infrequency indicator was created, where less-than-weekly social contact was considered as infrequent social contact, as has been the case in previous BHIS reports [57] and other studies [58, 59]. This has been measured since 1997. Functional social connection variables included perceived social support and social satisfaction. First, perceived social support was measured using the Oslo Social Support Scale (OSSS-3) [60]. It contains one item about the number of people on which one can count in case of a serious problem, one item about the degree of interest and concern close others (are perceived to) show and one item on the ease with which one can get help from a neighbor. The specific questions and response options can be found in SM 2. Following the scoring and cut-off guidelines of the OSSS-3, a total score was calculated, from which a binary perceived social support indicator was created, reflecting poor vs. moderate-to-strong social support [60]. This has been measured since 2008. Second, the survey contained a question on satisfaction with social contacts (see SM 2). From this question, a binary social satisfaction indicator was created, indicating either moderate to high social satisfaction (combining the response options “rather satisfying” and “very satisfying”) or low social satisfaction (combining the response options “rather unsatisfying” and “very unsatisfying”). This has been measured since 1997.

### Statistical analysis

In the descriptive analysis, the evolution of social connection was plotted over time, since the start of measurement of each social connection variable. This was done to help contextualize levels of and changes in social connection on a longer time scale. In addition, the 2023–2024 measurement was compared to the first available measurement, so that peri-pandemic changes can be evaluated in a broader context.

For the main analysis, all (binary) social connection indicators were analyzed using logistic regression models. Models were created using step-wise comparison of increasingly complex formulas, in which the most complex model that significantly improved model fit was selected. The fit of models was assessed using likelihood ratio tests, calculating the difference in -2 log-likelihoods between nested models. In all cases, this was the full model, which included year (i.e., 2018 vs. 2023–2024), age, sex, household type (i.e., living alone or not) and education level (i.e., non-tertiary education vs. tertiary education), as well as the interaction terms of the sociodemographic variables with year. Separate models were constructed for all social connection variables, including social frequency, social support and social satisfaction. For each indicator, the model was specified to test for the lack of social connection. This means the estimated event for social frequency was having infrequent social contacts (i.e., less than weekly), the estimated event for social support was the presence of poor social support and the estimated event for social satisfaction was the presence of low social satisfaction. All main analyses accounted for clustering (at the level of households) and stratification (at the level of regions, and health care regions or electoral districts), and were performed using the weighted dataset.

Stratified analyses were carried out to assess social connection in specific subgroups. The strata were created by selecting only those participants who met the subgroup condition (e.g., only those living alone for the stratified analyses in those living alone). Next, the logistic regression models were test in each separate stratum, including only those within the stratum who had responded to the social connection questions. The selected models were highly similar to the main model, always including year and all sociodemographic variables, except the one that was stratified on. The models also included interactions between year and all included sociodemographic variables. Because of the focus on the comparison of the pre- and post-COVID-19 waves, only main effects of year and interaction effects of sociodemographic variables with year will be discussed for the stratified models. Again, stratified analyses accounted for clustering (at the level of households) and stratification (at the level of regions, and health care regions or electoral districts), and were performed on the weighted dataset.

## Results

### Pre-post analyses

#### Frequency of social contacts

**Fig. 1:**
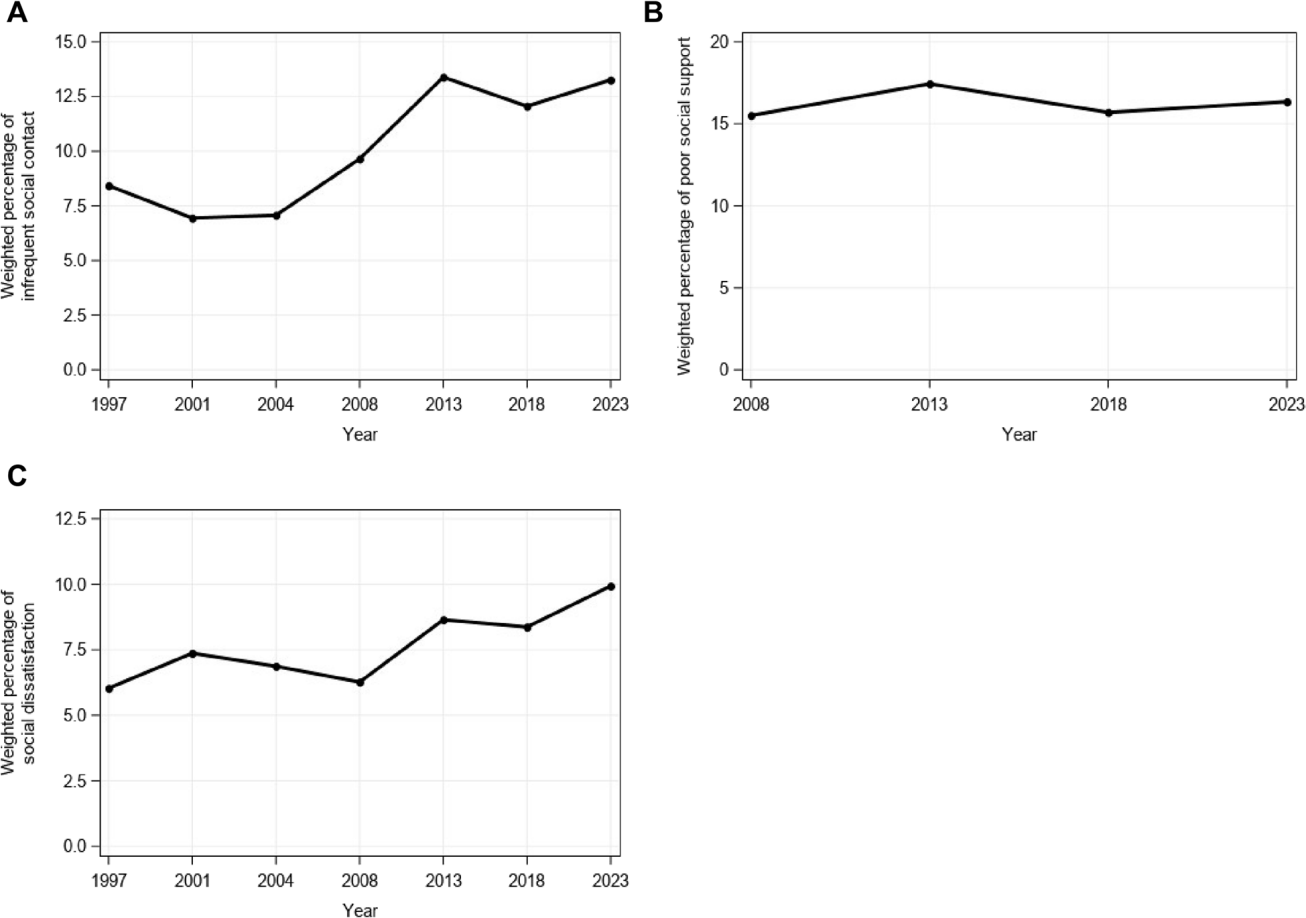
Evolution of social connection of the Belgian population aged 15 years and older. Note: (**A**) Evolution of the weighted percentage of the Belgian population aged 15 years and older that indicated having social contacts on a less than weekly basis, since the start of measurement in 1997. (**B**) Evolution of social support since the start of measurements in 2008. The graph shows the weighted percentage of poor social support over time. (**C**) Evolution of the weighted percentage of participants who indicated low satisfaction with social contacts in the past two weeks, since the start of measurement in 1997

Time trends in the percentage of participants with infrequent social contacts since the start of measurement in 1997 are shown in Fig. 1A to contextualize peri-pandemic changes on a longer time scale. This shows an increase in the percentage of participants indicating infrequent social contact since 1997 (*β* = 0.508, *SE* = 0.087, *p* < .001, *OR* = 1.662, 95% CI: 1.402, 1.969). However, this percentage seems to have stabilized in the past few waves, as no significant differences were seen between 2013, 2018 and 2023–2024 (all *p* > .098).

There was a main effect of education level, with higher odds of infrequent social contact in those without tertiary education (*β* = 0.447, *SE* = 0.103, *p* < .001, *OR* = 1.563, 95% CI: 1.277, 1.914), compared to those with tertiary education. Additionally, there was a main effect of household type, with higher odds of infrequent social contact in those living alone (*β* = 0.275, *SE* = 0.118, *p* = .019, *OR* = 1.317, 95% CI: 1.046, 1.658), compared to those not living alone. The main effect of sex was also significant, showing higher odds of infrequent social contacts in men (*β* = 0.284, *SE* = 0.088, *p* = .001, *OR* = 1.328, 95% CI: 1.118, 1.579) compared to women. Importantly, none of the other main effects or interaction effects between the sociodemographic factors and year were significant (all *p* > .296), meaning that changes over time were generally not significantly associated with certain sociodemographic factors.

#### Social support

Figure 1B shows the evolution of the percentage of poor social support since 2008 (i.e., start of measurement of social support), to contextualize peri-pandemic changes on a longer time scale. This shows stable levels when comparing 2018 and 2023–2024 (*p* = .085). These levels have not increased since 2008 (*p* = .374).

There was a main effect of education level, with higher odds of poor social support in those without tertiary education (*β* = 0.556, *SE* = 0.094, *p* < .001, *OR* = 1.743, 95% CI: 1.451, 2.094), compared to those with tertiary education. Additionally, there was a main effect of household type, with higher odds of poor social support in those living alone (*β* = 0.237, *SE* = 0.104, *p* = .023, *OR* = 1.267, 95% CI: 1.033, 1.554), compared to those not living alone. Again, none of the other main effects or interaction effects between the sociodemographic factors and year were significant (all *p* > .085).

#### Social satisfaction

The evolution of the percentage of the Belgian population aged 15 years and older indicating low social satisfaction is shown in Fig. 1C, to contextualize peri-pandemic changes on a longer time scale. This shows an increasing trend since the start of measurements in 1997, which was statistically significant (*β* = 0.541, *SE* = 0.093, *p* < .001, *OR* = 1.717, 95% CI: 1.432, 2.059). In the full model including socio-demographic variables, the odds of low social satisfaction were not significantly higher in 2023–2024 as compared to 2018 (*p* = .091).

There was a main effect of education level, with higher odds of low social satisfaction in those without tertiary education (*β* = 0.402, *SE* = 0.127, *p* = .002, *OR* = 1.494, 95% CI: 1.166, 1.915), compared to those with tertiary education. Additionally, there was a main effect of household type, with higher odds of low social satisfaction in those living alone (*β* = 0.560, *SE* = 0.126, *p* < .001, *OR* = 1.750, 95% CI: 1.367, 2.241), compared to those not living alone. The main effect of age was also significant, showing decreasing odds of low social satisfaction with increasing age (*β* = − 0.006, *SE* = 0.003, *p* = .046, *OR* = 0.994, 95% CI: 0.988, 0.999). None of the other main effects or interaction effects between sociodemographic factors and year were significant (all *p* > .091).

### Stratified analyses

Neither in women (*N* = 6844), nor in men (*N* = 6157) were there any significant main effects of year or interaction effects of the other sociodemographic variables with year (all *p* > .063).

**Fig. 2:**
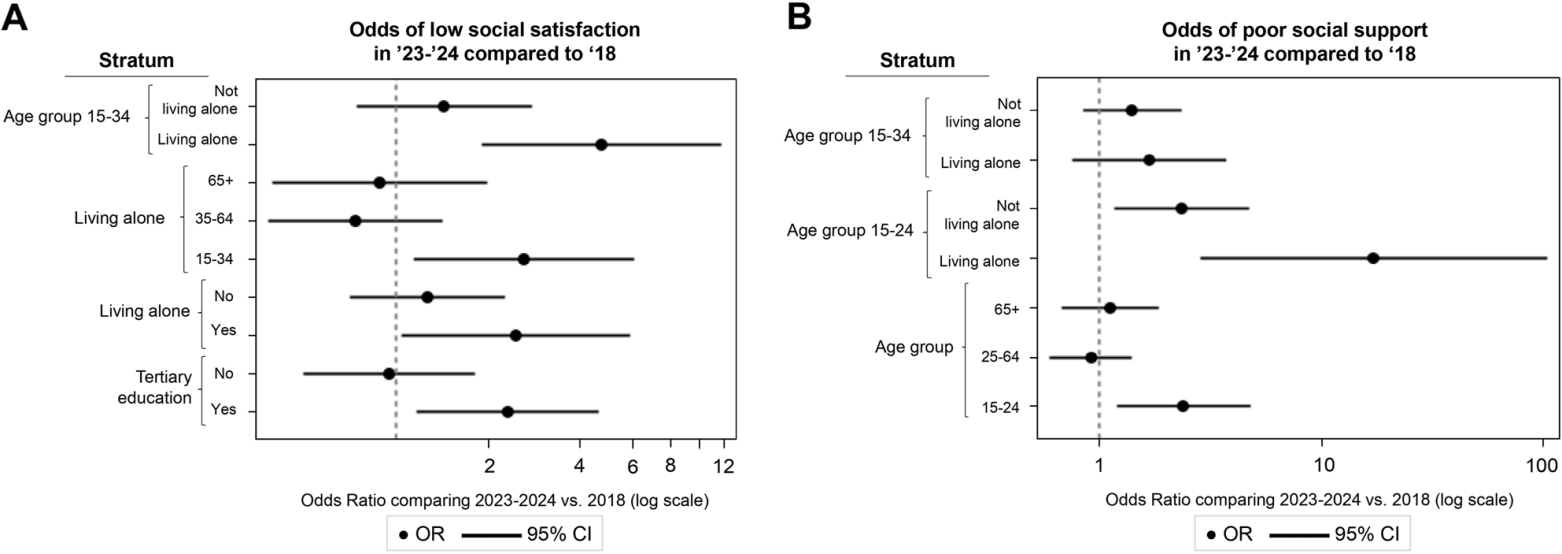
Social satisfaction and social support before (2018) versus after (2023–2024) the COVID-19 pandemic in substrata based on age, household situation and education. Note. (**A**) Year effects (main and interaction) of social satisfaction, comparing the odds of low social satisfaction in 2023 vs. 2018 in different strata and substrata. (**B**) Year effects (main and interaction) of social support, comparing the odds of poor social support in 2023 vs. 2018 in different strata and substrata

In participants with tertiary education (*N* = 5787), the odds of low social satisfaction were significantly higher in 2023 as compared to 2018 (*β* = 0.839, *SE* = 0.325, *p* = .017, *OR* = 2.315, 95% CI: 1.161, 4.615). This was also still the case when occupational status was additionally controlled for in post-hoc analyses. The effect is displayed in Fig. 2A. None of the other main or interaction effects with year were significant for any of the social connection indicators (all *p* > .078).

In participants living alone (*N* = 2995), the odds of low social satisfaction were significantly higher in 2023 compared to 2018 (*β* = 0.901, *SE* = 0.442, *p* = .042, *OR* = 2.461, 95% CI: 1.034, 5.859). In addition, there was an interaction effect between age group and year among those living alone (*β* = − 0.016, *SE* = 0.008, *p* = .045, *OR* = 0.985, 95% CI: 0.970, 0.999). Post-hoc analysis showed that this difference in social satisfaction between 2018 and 2023–2024 was only present in 15 to 24 year-olds living alone (*β* = 2.779, *SE* = 1.021, *p* = .007, *OR* = 16.099, 95% CI: 2.172, 119.33). However, since this is a very small group of participants (*N*_*2018*_ = 39, *N*_*2023−2024*_ = 14) and the odds ratio confidence interval is wide, this result should be interpreted with great caution. The same results are obtained when this analysis was repeated with a wider young age group (15 to 34 years), resulting in a larger sample of participants (N_2018_ = 253, *N*_*2023−2024*_ = 155) (*β* = 0.966, *SE* = 0.428, *p* = .024, *OR* = 2.628, 95% CI: 1.134, 6.087), still showing higher odds of low social satisfaction in 2023–2024 compared to 2018, only for 15 to 34 year-olds living alone, but not for those living alone aged 35 or older. The main effects of year on social support and social frequency among those living alone were not significant, and there were no significant interactions with year for any of the social connection indicators (all *p* > .077). For participants not living alone, there were no significant main effects of year or interaction effects with year for any of the social connection indicators (all *p* > .088). Figure 2A displays year effects of social satisfaction.

In participants aged 15 to 24 years old (*N* = 1102, of which 55 living alone), two indicators showed significant effects concerning year differences. First, although the main effect of year was not significant for social satisfaction (*p* = .278), there was a significant interaction between year and household type (*β* = 3.249, *SE* = 1.066, *p* = .002, *OR* = 25.753, 95% CI: 3.176, 208.820), with higher odds of low social satisfaction in 2023 (vs. 2018) in those living alone (*β* = 3.728, *SE* = 1.101, *p* = .001, *OR* = 41.609, 95% CI: 4.799, 360.780), but not in those not living alone (*p* = .277). However, since the group of 15- to 24-year-olds living alone who responded to the social satisfaction questions is very small (*N* = 53), these interactions should be interpreted with great caution. Additional analyses with a wider age group (i.e., 15 to 34 years old, *N* = 2728, of which 408 living alone, including only participants who responded to social satisfaction questions) showed the same interaction effect, with higher odds of low social satisfaction in 2023 vs. 2018 only in participants aged 15 to 34 years old living alone (*β* = 1.554, *SE* = 0.463, *p* = .001, *OR* = 4.730, 95% CI: 1.909, 11.722). These effects are also shown in Fig. 2A. Second, the odds of poor social support were significantly higher in 2023–2024 compared to 2018, which was particularly more pronounced in 15-to-24 year-olds living alone (*β* = 2.845, *SE* = 0.918, *p* = .002, *OR* = 17.195, 95% CI: 2.840, 104.120) than in those not living alone (*β* = 0.846, *SE* = 0.356, *p* = .018, *OR* = 2.331, 95% CI: 1.159, 4.689). However, this interaction should be interpreted with great caution due to the very small sample size of 15 to 24 year-olds living alone who responded to social support questions (*N* = 53). Post-hoc analyses in a wider and thus larger age group (i.e., 15 to 34 years old who responded to social support questions, *N* = 2728, of which 408 living alone) no longer showed a significant interaction between year and living situation (*p* = .65). These effects are shown in Fig. 2B. None of the social frequency effects of year were significant (all *p* > .193).

In 25-to-64 year-olds (*N* = 4074) and participants above 65 years old (*N* = 3543), there were no significant differences in social connection between 2018 and 2023–2024 (all *p* > .238). In addition, within these age groups, there were no interaction effects between the sociodemographic variables and year for any of the social connection indicators (all *p* > .107).

## Discussion

Since the COVID-19 pandemic, concerns have been raised over long-term changes in social connection that could be the result of enforced restrictions during the pandemic [61, 62]. In response to this, the current study assessed structural and functional social connection in the peri-pandemic period, comparing pre-pandemic (i.e., 2018) and post-pandemic (i.e., 2023–2024) levels of social connection in the general Belgian population of 15 years and older, and in specific socio-demographic subgroups.

### Social connection before versus after the pandemic

In the general population, no evidence was found for long-term decreases in social connection since the COVID-19 pandemic. Between 2018 and 2023–2024, there were no significant differences in structural social connection (i.e., frequency of social contacts). This is not in line with H1 or with findings of earlier studies [28, 29], in which structural social connection had not fully recovered to pre-pandemic levels. However, in these studies post-pandemic measurements date from 2022 to May 2023, whereas the current study used data from 2023 to 2024. It is possible that more recovery has taken place over time, and thus, that the longer time frame of the current study explains the conflicting findings. There were also no general differences between 2018 and 2023–2024 in functional social connection (i.e., social satisfaction and social support). This is in line with H2 and the findings of van Tilburg [31], who observed similar levels of functional social connection when comparing pre- and post-pandemic measurements (i.e., in 2023) in the Netherlands. However, this finding is not in line with the findings of Poštuvan et al. [30], who observed higher levels of loneliness after the pandemic (i.e., in 2023) in Slovenia. Interestingly, the same instrument was used to measure loneliness (DJG-6) in both studies. However, there were differences in recruitment strategies, and these studies were carried out in different countries. It has been shown before that there was variation between countries in the severity of the COVID-19 crisis [63], the stringency of policies to tackle it [64, 65], and its effects on outcomes such as mental health [66]. Thus, it is possible that the effect of the COVID-19 pandemic on social connection varies among countries. Future research could compare countries and investigate whether variables such as excess deaths and policy stringency predict social connection. In any case, it can be seen as reassuring that no clear increases in the prevalence of poor structural and functional social connection were observed in the general Belgian population.

In certain specific socio-demographic subgroups, however, there were peri-pandemic differences in social connection levels, partially in line with H3. Stratified analyses showed significant differences within the age group of the 15- to 24-year-olds. They had significantly higher odds of poor social support in 2023–2024 as compared to 2018, but no such effect was present for social satisfaction or social contact frequency. This is partially in line with the concerns that were raised specifically over the social well-being of adolescents and young adults during the COVID-19 pandemic. It is also in line with previous studies that have showed decreased social support and increased loneliness and mental health issues in adolescents and young adults during the COVID-19 pandemic [67–71].

A similar pre-post difference was observed within the stratum of those with tertiary education. Within this group, the odds of low social satisfaction were higher in 2023–2024 as compared to 2018. As far as we know, no such result has been observed in the currently still relatively limited literature on social connection in the context of COVID-19 in specific subgroups. Neither of the aforementioned longer-term studies reported having analyzed education [30, 31]. Additional post-hoc analyses showed that this pre-post difference could not be explained by employment status. However, another alternative explanation could be the rise in teleworking since the COVID-19 pandemic [72, 73], which has particularly affected the highly educated [72, 74]. Although there are advantages to teleworking, it might also be associated with loneliness [75]. While this is a plausible interpretation, teleworking information is currently not available in the BHIS, and so, additional research would be needed for further clarification. Additionally, it should be noted that the main effect of education found in the total population remains important as well, showing that, generally speaking, those without tertiary education have a higher prevalence of poor social connection than those with tertiary education (even if the latter has seen an increase in the prevalence of poor social connection over time).

Finally, the odds of low social satisfaction were also higher after the pandemic compared to before in the stratum of those living alone, which was particularly pronounced in younger participants. Since individuals living alone rely on contact outside of the home for social connection and the majority of the social restrictions mostly affected out-of-household contact, a short-term impact would be expected, but observing this in 2023–2024 suggests a potential long-term impact on social connection. This raises the question whether the COVID-19 pandemic has affected social relationship formation and maintenance, which are developmentally particularly important in adolescence and young adulthood. However, whereas living alone has been identified a risk factor for loneliness in general [33] as well as during the pandemic [32], no other longer-term studies including living arrangements are available yet for comparison. Studies on peri- and post-pandemic social relationship formation and maintenance are also yet to emerge.

### Social inequalities

These findings show that there are social inequalities in social connection, both in general, as seen in main effects in the general population analyses, and in the context of the COVID-19 pandemic, as seen in year effects in certain stratified analyses. The main effects found in the general population analysis have consistently shown worse social connection in those living alone (compared to those not living alone) and those without tertiary education (compared to those with tertiary education), for all three tested social connection indicators. This is in line with previous research on social inequalities in social connection [76], and in health in general [77–79]. For those living alone, it is particularly concerning that they showed poorer social connection in general (i.e., main effect in the main analyses), as well as decreased social connection when comparing pre- and post-pandemic levels (i.e., year effect within the stratum of those living alone). This stresses the importance of developing interventions and policies that are particularly directed towards and sensitive of those living alone. Regarding inequalities between individuals with different levels of education, it is important to note that the general level of social connection remains lower in those without tertiary education compared to those with tertiary education, even if a decline in social satisfaction has been observed within those with tertiary education when comparing pre- and post-pandemic levels. Two considerations are noteworthy. First, the decline of social satisfaction levels within those with tertiary education is worth further investigation, for example by exploring the role of teleworking. Second, it remains important to include those without tertiary education in socially sensitive interventions and policies.

Addressing these social inequalities is important because of the long-term consequences of poor social connection, which may translate in long-term social inequalities in mental and physical health. After all, it has been shown that poor social connection is a strong risk factor for the development of several diseases, mental health problems and mortality [80]. In addition, as this influence is assumed to be bidirectional, future research could also assess the role of pre-existing health conditions (e.g., chronic illnesses such as asthma) in shaping social connection.

### Strengths and limitations

Taken together, this study shows that there are no longer-term declines in social connection in the general Belgian population since the COVID-19 pandemic. However, a long-term impact might be present for adolescents and young adults, and those living alone. With this long-term focus, we are complementing the existing literature on the short-term consequences of the COVID-19 pandemic. Using a combination of large-scale registry-based sampling and post-stratification weighting, we were able to obtain population-representative estimates and evaluate changes in structural and functional social connection on a national level. The availability of pre-pandemic waves in the BHIS also allowed for consistent comparisons across time. The subgroup analyses allowed us to identify specific risk factors for long-term consequences of a pandemic on social connection.

Some limitations have to be considered, however. First, since the current study used cross-sectional, and not longitudinal data, within-person changes could not be evaluated. Consequently, it was also not possible to study the predictors of deteriorated social connection. However, the current study does allow for the estimation of the national population prevalence of social connection indicators, and group-level shifts in these indicators. Second, even though the pre-post comparison allows for a long-term comparison of population prevalence and odds, the evaluation of social connection in the context of the COVID-19 pandemic would have ideally also involved a measurement during the most acute phase of the pandemic. However, the current data collection methodology was impossible in the context of a pandemic. Therefore, these results can be compared to other short-term studies (i.e., including measurements before and during the pandemic), albeit with awareness for methodological and contextual heterogeneity. Third, our measure for structural social connection (i.e., frequency of social contacts) consisted of only one question, whereas more detailed measures (e.g., social contact diaries, reporting specific social contacts per day) have been used in other studies [18, 28, 29], making our indicator potentially less sensitive. This lack of detail in our structural social connection is an alternative explanation for the lack of peri-pandemic changes in structural social connection in this study, as changes may have been only present on the scale of daily to weekly social contacts, whereas the most frequent response option in our question was “at least once per week”. The measure was used as it was already present in the BHIS and ensured comparison across waves. Similarly, our social satisfaction indicator is based on a single, general question, whereas most studies have used more clearly defined constructs and validated instruments for functional social connection [23, 24, 30, 31]. It is possible that we found no changes in social satisfaction, whereas previous studies did find decreases in functional social connection, due to methodological differences. It would have been better to use a loneliness indicator, but this was not available yet in 2018. However, considering that social support was measured using a validated instrument, and that the findings on social support in 15 to 24 year-olds are consistent with the current literature, there seems to be an indication of negatively impacted functional social connection in this age group. Fourth, although the (repeated cross-sectional) pre- vs. post-pandemic social support difference observed in 15- to 24-year-olds and those living alone could be a direct consequence of measures enforced during the COVID-19 pandemic, it could also be caused by the pandemic only indirectly (e.g., due to other changes induced by the pandemic), or even not at all (e.g., an unrelated generation effect). The current data and methodology do not allow us to make causal claims, and thus only provide indications. Fifth, due to very small subsample sizes, the interaction effects tested in the stratified analyses involving 15- to 24-year-olds living alone should be interpreted with great caution. Additional analyses in a wider, and thus larger, age group (15 to 34 years old) showed that this effect remained present for social satisfaction but not for social support. However, still, the main focus in the interpretation of the results should lie with the difference observed in the total stratum of 15- to 24-year-olds (i.e., higher odds of social connection problems in 2023–2024 vs. 2018). Nevertheless, the findings showing interactions within strata may provide a basis for hypothesis development in future studies using larger samples. Sixth, while the use of binary indicators favors simplicity of interpretation and helps to maintain more adequate subsample sizes, especially in the stratified analyses, this can also lead to information loss and oversimplification, potentially masking finer nuances in the data. To address this limitation, we repeated the main analyses with non-dichotomized versions of the same social connection indicators. The results of these analyses (see SM 3) were highly in line with the main (binary) analyses. The most important difference is that, indeed, a finer nuance was found for social satisfaction. Whereas there was no pre- vs. post-pandemic difference in the percentage of participants in the general population who indicated to be socially satisfied, there was a significant shift in the distribution of the “very satisfied” and “rather satisfied” responses, in which “very satisfied” became less prevalent, and “rather satisfied” became more prevalent after the pandemic. This suggests a subtle post-pandemic change in social satisfaction that invites follow-up research. Seventh, while including different indicators that each capture different components of social connection and studying this in different subgroups is highly socially relevant, this also increases the risk of chance findings.

## Conclusion

In conclusion, both structural and functional social connection showed similar pre- and post-COVID-19 levels in the general Belgian population. However, those living alone, those with tertiary education, and adolescents and young adults showed higher odds of poor functional social connection after the pandemic, as compared to before. While no causal claims can be made on this basis and alternative explanations exist, these findings do suggest that certain subgroups might have suffered more from the COVID-19 pandemic than other groups. Our findings highlight these groups as relevant for (longitudinal) follow-up research. Evaluating longer-term social connection levels is highly relevant in light of our preparedness for future pandemics, particularly when weighing the benefits of certain preventive measures with their potential long-term consequences. Our results show that some subgroups in the population might have been more vulnerable in terms of social connection, and argue for heightened awareness for and additional research in the young and those living alone.

## Supplementary Information

Supplementary Material.

## Data Availability

Access to the data reported this article can be granted on reasonable request to HIS@sciensano.be.

## Abbreviations

COVID-19: Coronavirus disease 2019
BHIS: Belgian Health Interview Survey
OSSS-3: Oslo Social Support Scale (3-item version)
H: Hypothesis (numbered)
SM: Supplementary material (numbered)

## References

[1] Ayouni I, Maatoug J, Dhouib W, Zammit N, Fredj SB, Ghammam R, et al. Effective public health measures to mitigate the spread of COVID-19: a systematic review. BMC Public Health. 2021;21(1015). 10.1186/s12889-021-11111-1. 10.1186/s12889-021-11111-1 PMC8164261 34051769

[2] Ngonghala CN, Iboi E, Eikenberry S, Scotch M, MacIntyre CR, Bonds MH, et al. Mathematical assessment of the impact of non-pharmaceutical interventions on curtailing the 2019 novel Coronavirus. Math Biosci. 2020;325:108364. 10.1016/j.mbs.2020.108364. 32360770 10.1016/j.mbs.2020.108364 PMC7252217

[3] Talic S, Shah S, Wild H, Gasevic D, Maharaj A, Ademi Z, et al. Effectiveness of public health measures in reducing the incidence of covid-19, SARS-CoV-2 transmission, and covid-19 mortality: systematic review and meta-analysis. BMJ. 2021;375:e068302. 10.1136/bmj-2021-068302. 34789505 10.1136/bmj-2021-068302 PMC9423125

[4] Ducharme J. COVID-19 Is Making America’s Loneliness Epidemic Even Worse. Time Magazine. 2020. Available from: https://time.com/5833681/loneliness-covid-19/.

[5] Gabbatt A. ‘Social recession’: how isolation can affect physical and mental health. The Guardian. 2020. Available from: https://www.theguardian.com/world/2020/mar/18/coronavirus-isolation-social-recession-physical-mental-health.

[6] Psychiater Dirk De Wachter waarschuwt. Na de piek van het coronavirus, komt de piek van de psychologische problemen. VRT NWS. 2020 Apr 2. Available from: https://www.vrt.be/vrtnws/nl/2020/04/02/psychiater-dirk-de-wachter-coronavirus-quarantaine/.

[7] Killgore WDS, Cloonan SA, Taylor EC, Dailey NS, Loneliness. A signature mental health concern in the era of COVID-19. Psychiatry Res. 2020;290:113117. 10.1016/j.psychres.2020.113117. 32480121 10.1016/j.psychres.2020.113117 PMC7255345

[8] Baarck J, d’Hombres B, Tintori G. Loneliness in Europe before and during the COVID-19 pandemic. Health Policy. 2022;126(11):1124–9. 36182348 10.1016/j.healthpol.2022.09.002 PMC9479375

[9] Wickramaratne PJ, Yangchen T, Lepow L, Patra BG, Glicksburg B, Talati A, et al. Social connectedness as a determinant of mental health: a scoping review. Pan X, editor. PLOS ONE. 2022;17(10):e0275004. 10.1371/journal.pone.0275004. 10.1371/journal.pone.0275004 PMC9560615 36228007

[10] Holt-Lunstad J, Smith TB, Baker M, Harris T, Stephenson D. Loneliness and Social Isolation as Risk Factors for Mortality: A Meta-Analytic Review. Perspect Psychol Sci. 2015;10(2):227–37. 10.1177/1745691614568352. 25910392 10.1177/1745691614568352

[11] Holt-Lunstad J. Social Connection as a Public Health Issue: The Evidence and a Systemic Framework for Prioritizing the Social in Social Determinants of Health. Annu Rev Public Health. 2022;43(1):193–213. 10.1146/annurev-publhealth-052020-110732. 35021021 10.1146/annurev-publhealth-052020-110732

[12] Holt-Lunstad J. Why Social Relationships Are Important for Physical Health: A Systems Approach to Understanding and Modifying Risk and Protection. Annu Rev Psychol. 2018;69(1):437–58. 10.1146/annurev-psych-122216-011902. 29035688 10.1146/annurev-psych-122216-011902

[13] Holt-Lunstad J, Steptoe A. Social isolation: An underappreciated determinant of physical health. Curr Opin Psychol. 2022;43:232–7. 10.1016/j.copsyc.2021.07.012. 34438331 10.1016/j.copsyc.2021.07.012

[14] Taylor HO. Social Isolation’s Influence on Loneliness Among Older Adults. Clin Soc Work J. 2020;48(1):140–51. 10.1007/s10615-019-00737-9. 33343042 10.1007/s10615-019-00737-9 PMC7747874

[15] de Jong-Gierveld J, van Tilburg TG, Dykstra PA. Loneliness and Social Isolation. The Cambridge Handbook of Personal Relationships. Cambridge University Press; 2006. pp. 485–500.

[16] Rollier M, Miranda GHB, Vergeynst J, Meys J, Alleman TW, Baetens JM. Mobility and the spatial spread of sars-cov-2 in Belgium. Math Biosci. 2023;360:108957. 10.1016/j.mbs.2022.108957. 36804448 10.1016/j.mbs.2022.108957 PMC9934928

[17] Liu CY, Berlin J, Kiti MC, Del Fava E, Grow A, Zagheni E, et al. Rapid Review of Social Contact Patterns During the COVID-19 Pandemic. Epidemiology. 2021;32(6):781–91. 10.1097/EDE.0000000000001412. 34392254 10.1097/EDE.0000000000001412 PMC8478104

[18] Drolet M, Godbout A, Mondor M, Béraud G, Drolet-Roy L, Lemieux-Mellouki P, et al. Time trends in social contacts before and during the COVID-19 pandemic: the CONNECT study. BMC Public Health. 2022;22(1):1032. 10.1186/s12889-022-13402-7. 35606703 10.1186/s12889-022-13402-7 PMC9125550

[19] Peng S, Roth AR. Social Isolation and Loneliness Before and During the COVID-19 Pandemic: A Longitudinal Study of U.S. Adults Older Than 50. Kelley J, editor. J Gerontol Ser B. 2022;77(7):e185–90. 10.1093/geronb/gbab068. 10.1093/geronb/gbab068 PMC8083229 33870414

[20] Murayama H, Okubo R, Tabuchi T. Increase in Social Isolation during the COVID-19 Pandemic and Its Association with Mental Health: Findings from the JACSIS 2020 Study. Int J Environ Res Public Health. 2021;18(16):8238. 10.3390/ijerph18168238. 34443988 10.3390/ijerph18168238 PMC8394951

[21] Ernst M, Niederer D, Werner AM, Czaja SJ, Mikton C, Ong AD, et al. Loneliness before and during the COVID-19 pandemic: A systematic review with meta-analysis. Am Psychol. 2022;77(5):660–77. 10.1037/amp0001005. 35533109 10.1037/amp0001005 PMC9768682

[22] Buecker S, Horstmann KT. Loneliness and social isolation during the COVID-19 pandemic: A systematic review enriched with empirical evidence from a large-scale diary study. Eur Psychol. 2021;26(4):272–84. 10.31234/osf.io/3dwxq.

[23] Luchetti M, Lee JH, Aschwanden D, Sesker A, Strickhouser JE, Terracciano A, et al. The trajectory of loneliness in response to COVID-19. Am Psychol. 2020;75(7):897–908. 10.1037/amp0000690. 32567879 10.1037/amp0000690 PMC7890217

[24] Philpot LM, Ramar P, Roellinger DL, Barry BA, Sharma P, Ebbert JO. Changes in social relationships during an initial stay-at-home phase of the COVID-19 pandemic: A longitudinal survey study in the U.S. Soc Sci Med. 2021;274:113779. 10.1016/j.socscimed.2021.113779. 33639395 10.1016/j.socscimed.2021.113779 PMC7895700

[25] Kourti A, Stavridou A, Panagouli E, Psaltopoulou T, Spiliopoulou C, Tsolia M, et al. Domestic Violence During the COVID-19 Pandemic: A Systematic Review. Trauma Violence Abuse. 2023;24(2):719–45. 10.1177/15248380211038690. 34402325 10.1177/15248380211038690 PMC10011925

[26] Drieskens S, Braekman E, Ridder KD, Gisle L, Charafeddine R, Hermans L, et al. Domestic violence during the COVID-19 confinement: do victims feel more socially isolated? Arch Public Health. 2022;80(1):39. 10.1186/s13690-021-00765-3. 35078519 10.1186/s13690-021-00765-3 PMC8787181

[27] Schokkenbroek JM, Hardyns W, Anrijs S, Ponnet K. Partners in lockdown: Relationship stress in men and women during the COVID-19 pandemic. Couple Fam Psychol Res Pract. 2021;10(3):149–57. 10.1037/cfp0000172.

[28] Jarvis CI, Coletti P, Backer JA, Munday JD, Faes C, Beutels P, et al. Social contact patterns following the COVID-19 pandemic: a snapshot of post-pandemic behaviour from the CoMix study. Epidemics. 2024;48. 10.1016/j.epidem.2024.100778. 10.1016/j.epidem.2024.100778 PMC11413520 38964131

[29] Backer JA, Vos ERA, Den Hartog G, Van Hagen CCE, De Melker HE, Van Der Klis FRM, et al. Contact behaviour before, during and after the COVID-19 pandemic in the Netherlands: evidence from contact surveys, 2016 to 2017 and 2020 to 2023. Eurosurveillance. 2024;29(43). 10.2807/1560-7917.ES.2024.29.43.2400143. 10.2807/1560-7917.ES.2024.29.43.2400143 PMC11513762 39450517

[30] Poštuvan V, Krohne N, Lavrič M, Gomboc V, De Leo D, Rojs L. A Lonelier World after COVID-19: Longitudinal Population-Based Study of Well-Being, Emotional and Social Loneliness, and Suicidal Behaviour in Slovenia. Med (Mex). 2024;60(2):312. 10.3390/medicina60020312. 10.3390/medicina60020312 PMC10890292 38399599

[31] Van Tilburg TG. Residual loneliness in the Netherlands after the COVID-19 pandemic: An application of the single interrupted time series design with pre-, peri- and post-pandemic observations. Public Health. 2024;237:238–44. 10.1016/j.puhe.2024.10.023. 39461031 10.1016/j.puhe.2024.10.023

[32] Bu F, Steptoe A, Fancourt D. Who is lonely in lockdown? Cross-cohort analyses of predictors of loneliness before and during the COVID-19 pandemic. Public Health. 2020;186:31–4. 10.1016/j.puhe.2020.06.036. 32768621 10.1016/j.puhe.2020.06.036 PMC7405905

[33] Barjaková M, Garnero A, d’Hombres B. Risk factors for loneliness: A literature review. Soc Sci Med. 2023;334:116163. 10.1016/j.socscimed.2023.116163. 37625251 10.1016/j.socscimed.2023.116163 PMC10523154

[34] Seifert A, Hassler B. Impact of the COVID-19 Pandemic on Loneliness Among Older Adults. Front Sociol. 2020;5:590935. 10.3389/fsoc.2020.590935. 33869519 10.3389/fsoc.2020.590935 PMC8022464

[35] Somerville LH. The Teenage Brain: Sensitivity to Social Evaluation. Curr Dir Psychol Sci. 2013;22(2):121–7. 10.1177/0963721413476512. 24761055 10.1177/0963721413476512 PMC3992953

[36] Farrell AH, Vitoroulis I, Eriksson M, Vaillancourt T. Loneliness and Well-Being in Children and Adolescents during the COVID-19 Pandemic: A Systematic Review. Children. 2023;10(2):279. 10.3390/children10020279. 36832408 10.3390/children10020279 PMC9955087

[37] Houghton S, Kyron M, Hunter SC, Lawrence D, Hattie J, Carroll A, et al. Adolescents’ longitudinal trajectories of mental health and loneliness: The impact of COVID-19 school closures. J Adolesc. 2022;94(2):191–205. 10.1002/jad.12017. 35353417 10.1002/jad.12017 PMC9087620

[38] Orben A, Tomova L, Blakemore SJ. The effects of social deprivation on adolescent development and mental health. Lancet Child Adolesc Health. 2020;4(8):634–40. 10.1016/S2352-4642(20)30186-3. 32540024 10.1016/S2352-4642(20)30186-3 PMC7292584

[39] Picton C, Kahu ER, Nelson K. Friendship supported learning – the role of friendships in first-year students’ university experiences.

[40] Alotaibi TA, Alkhalifah KM, Alhumaidan NI, Almutiri WA, Alsaleh SK, AlRashdan FM, et al. The benefits of friendships in academic settings: a systematic review and meta-analysis. Cureus. 2023. 10.7759/cureus.50946. 10.7759/cureus.50946 PMC10800095 38249290

[41] Kulcar V, Bork-Hüffer T, Schneider AM. Getting Through the Crisis Together: Do Friendships Contribute to University Students’ Resilience During the COVID-19 Pandemic? Front Psychol. 2022;13:880646. 10.3389/fpsyg.2022.880646. 35651553 10.3389/fpsyg.2022.880646 PMC9149295

[42] Weber M, Schulze L, Bolzenkötter T, Niemeyer H, Renneberg B. Mental Health and Loneliness in University Students During the COVID-19 Pandemic in Germany: A Longitudinal Study. Front Psychiatry. 2022;13:848645. 10.3389/fpsyt.2022.848645. 35492687 10.3389/fpsyt.2022.848645 PMC9051079

[43] Werner AM, Tibubos AN, Mülder LM, Reichel JL, Schäfer M, Heller S, et al. The impact of lockdown stress and loneliness during the COVID-19 pandemic on mental health among university students in Germany. Sci Rep. 2021;11(1):22637. 10.1038/s41598-021-02024-5. 34811422 10.1038/s41598-021-02024-5 PMC8609027

[44] Wu B. Social isolation and loneliness among older adults in the context of COVID-19: a global challenge. Glob Health Res Policy. 2020;5(1):27. 10.1186/s41256-020-00154-3. 32514427 10.1186/s41256-020-00154-3 PMC7272234

[45] Su Y, Rao W, Li M, Caron G, D’Arcy C, Meng X. Prevalence of loneliness and social isolation among older adults during the COVID-19 pandemic: A systematic review and meta-analysis. Int Psychogeriatr. 2023;35(5):229–41. 10.1017/S1041610222000199. 35357280 10.1017/S1041610222000199

[46] Shin H, Park C. Gender differences in social networks and physical and mental health: are social relationships more health protective in women than in men? Front Psychol. 2023;14:1216032. 10.3389/fpsyg.2023.1216032. PubMed PMID: 38213610; PubMed Central PMCID: PMC10782512. 38213610 10.3389/fpsyg.2023.1216032 PMC10782512

[47] Umberson D, Lin Z, Cha H. Gender and Social Isolation across the Life Course. J Health Soc Behav. 2022;63(3):319–35. doi:10.1177/00221465221109634 PubMed PMID: 35856404; PubMed Central PMCID: PMC10409601. 35856404 10.1177/00221465221109634 PMC10409601

[48] De Jong Gierveld J, Van Tilburg T. The De Jong Gierveld short scales for emotional and social loneliness: tested on data from 7 countries in the UN generations and gender surveys. Eur J Ageing. 2010;7(2):121–30. 10.1007/s10433-010-0144-6. 20730083 10.1007/s10433-010-0144-6 PMC2921057

[49] Fierloos IN, Tan SS, Williams G, Alhambra-Borrás T, Koppelaar E, Bilajac L, et al. Socio-demographic characteristics associated with emotional and social loneliness among older adults. BMC Geriatr. 2021;21(1):114. 10.1186/s12877-021-02058-4. 33563228 10.1186/s12877-021-02058-4 PMC7871533

[50] Beller J. Social inequalities in loneliness: disentangling the contributions of education, income, and occupation. Sage Open. 2024;14(3). 10.1177/21582440241281408.

[51] Zhuo J, Harrigan N. Low education predicts large increase in COVID-19 mortality: the role of collective culture and individual literacy. Public Health. 2023;221:201–7. 10.1016/j.puhe.2023.06.016. 37487422 10.1016/j.puhe.2023.06.016 PMC10284448

[52] McGowan VJ, Bambra C. COVID-19 mortality and deprivation: pandemic, syndemic, and endemic health inequalities. Lancet Public Health. 2022;7(11):e966–75. 10.1016/S2468-2667(22)00223-7. 36334610 10.1016/S2468-2667(22)00223-7 PMC9629845

[53] Khetan AK, Yusuf S, Lopez-Jaramillo P, Szuba A, Orlandini A, Mat-Nasir N, et al. Variations in the financial impact of the COVID-19 pandemic across 5 continents: a cross-sectional, individual level analysis. eClinicalMedicine. 2022;44. 10.1016/j.eclinm.2022.101284. PubMed PMID: 35106472. 10.1016/j.eclinm.2022.101284 PMC8794545 35106472

[54] Demarest S, Van Der Heyden J, Charafeddine R, Drieskens S, Gisle L, Tafforeau J. Methodological basics and evolution of the Belgian health interview survey 1997–2008. Arch Public Health. 2013;71(1):24. 10.1186/0778-7367-71-24. 24047278 10.1186/0778-7367-71-24 PMC3844891

[55] Demarest S, Charafeddine R. Report: D/2025.14.440/29. Brussels, Belgium: Sciensano; 2025. Available from: www.gezondheidsenquete.be. Gezondheidsenquête 2023–2024: Methodologie.

[56] Association WM. World Medical Association Declaration of Helsinki: Ethical Principles for Medical Research Involving Human Participants. JAMA. 2025;333(1):71–4. 10.1001/jama.2024.21972. 39425955 10.1001/jama.2024.21972

[57] Braekman E, Berete F, Drieskens S, Charafeddine RGezondheidsenquête. 2018: Sociale gezondheid.. Brussels, Belgium: Sciensano. Report: D/2020/14.440/57. Available from: www.gezondheidsenquête.be.

[58] Choi KW, Waite LJ, Finch LE, Kotwal AA. Social Isolation and Worsening Health Behaviors Among Older Adults During the COVID-19 Pandemic. J Gerontol B Psychol Sci Soc Sci. 2023;78(11):1903–16. 10.1093/geronb/gbad122. PubMed PMID: 37591797; PubMed Central PMCID: PMC10645306. 37591797 10.1093/geronb/gbad122 PMC10645306

[59] Sakurai R, Yasunaga M, Nishi M, Fukaya T, Hasebe M, Murayama Y, et al. Co-existence of social isolation and homebound status increase the risk of all-cause mortality. Int Psychogeriatr. 2019;31(5):703–11. 10.1017/S1041610218001047. 30022745 10.1017/S1041610218001047

[60] Kocalevent RD. Social support in the general population: standardization of the Oslo social support scale (OSSS-3). BMC Psychol. 2018;6(31). 10.1186/s40359-018-0249-9. 10.1186/s40359-018-0249-9 PMC6050647 30016997

[61] Holmes EA, O’Connor RC, Perry VH, Tracey I, Wessely S, Arseneault L, et al. Multidisciplinary research priorities for the COVID-19 pandemic: a call for action for mental health science. Lancet Psychiatry. 2020;7(6):547–60. 10.1016/S2215-0366(20)30168-1. 32304649 10.1016/S2215-0366(20)30168-1 PMC7159850

[62] Wright R. How Loneliness from Coronavirus Isolation Takes Its Own Toll. The New Yorker. 2020. Available from: https://www.newyorker.com/news/our-columnists/how-loneliness-from-coronavirus-isolation-takes-its-own-toll.

[63] Msemburi W, Karlinsky A, Knutson V, Aleshin-Guendel S, Chatterji S, Wakefield J. The WHO estimates of excess mortality associated with the COVID-19 pandemic. Nature. 2023;613(7942):130–7. 10.1038/s41586-022-05522-2. 36517599 10.1038/s41586-022-05522-2 PMC9812776

[64] Balmford B, Annan JD, Hargreaves JC, Altoè M, Bateman IJ. Cross-Country Comparisons of Covid-19: Policy, Politics and the Price of Life. Environ Resour Econ. 2020;76(4):525–51. 10.1007/s10640-020-00466-5. 10.1007/s10640-020-00466-5 PMC7400753 32836862

[65] Chazel L. Stay at Home! A Comparative Analysis of the Implementation of Lockdowns as a Response to the COVID-19 Pandemic. In: Egger C, Magni-Berton R, de Saint-Phalle E, editors. Covid-19 Containment Policies in Europe. Cham: Springer Nature Switzerland; 2024 [cited 2025 Jun 16]. pp. 209–20. Available from: 10.1007/978-3-031-52096-9_12.

[66] Aknin LB, Andretti B, Goldszmidt R, Helliwell JF, Petherick A, Neve JED, et al. Policy stringency and mental health during the COVID-19 pandemic: a longitudinal analysis of data from 15 countries. Lancet Public Health. 2022;7(5):e417–26. 10.1016/S2468-2667(. 22)00060-3 PubMed PMID: 35461592. 35461592 10.1016/S2468-2667(22)00060-3 PMC9023007

[67] Schütz R, Bilz L. Increasing loneliness among German children and adolescents from 2018 to 2022: a cross-sectional survey before and after the onset of the COVID-19 pandemic. J Public Health. 2024;23. 10.1007/s10389-024-02356-2.

[68] Lee CM, Cadigan JM, Rhew IC. Increases in Loneliness Among Young Adults During the COVID-19 Pandemic and Association With Increases in Mental Health Problems. J Adolesc Health. 2020;67(5):714–7. 10.1016/j.jadohealth.2020.08.009. 33099414 10.1016/j.jadohealth.2020.08.009 PMC7576375

[69] Hawes MT, Szenczy AK, Klein DN, Hajcak G, Nelson BD. Increases in depression and anxiety symptoms in adolescents and young adults during the COVID-19 pandemic. Psychol Med. 2022;52(14):3222–30. 10.1017/S0033291720005358. 33436120 10.1017/S0033291720005358 PMC7844180

[70] Robinson E, Sutin AR, Daly M, Jones A. A systematic review and meta-analysis of longitudinal cohort studies comparing mental health before versus during the COVID-19 pandemic in 2020. J Affect Disord. 2022;296:567–76. 10.1016/j.jad.2021.09.098. 34600966 10.1016/j.jad.2021.09.098 PMC8578001

[71] Magis-Weinberg L, Arreola Vargas M, Carrizales A, Trinh CT, Muñoz Lopez DE, Hussong AM, et al. The impact of COVID‐19 on the peer relationships of adolescents around the world: A rapid systematic review. J Res Adolesc. 2025;35(1):e12931. 10.1111/jora.12931. 38682766 10.1111/jora.12931

[72] Vargas Llave O, Hurley J, Peruffo E, Rodríguez R, Adascalitei D, Botey Gaude L, et al. The rise in telework: impact on working conditions and regulations: working conditions. Luxembourg: Publications Office of the European Union; 2022. Report. 10.2806/069206.

[73] Soler JRL, Christidis P, Vassallo JM. Evolution of teleworking and urban mobility changes driven by the COVID-19 pandemic across European Cities. Transp Res Procedia. 2023;AIIT 3rd International Conference on Transport Infrastructure and Systems (TIS ROMA 2022), 15th-16th September 2022, Rome, Italy. 69:488–95. 10.1016/j.trpro.2023.02.199.

[74] Brussevich M, Dabla-Norris E, Khalid S. Who Bears the Brunt of Lockdown Policies? Evidence from Tele-workability Measures Across Countries. IMF Econ Rev. 2022;70(3):560–89. 10.1057/s41308-022-00165-9.

[75] Fostervold KI, Ulleberg P, Nilsen OV, Halberg AM. The hidden costs of working from home: examining loneliness, role overload, and the role of social support during and beyond the COVID-19 lockdown. Front Organ Psychol. 2024;2. 10.3389/forgp.2024.1380051.

[76] Hutten E, Jongen EMM, Hajema K, Ruiter RAC, Hamers F, Bos AER. Risk factors of loneliness across the life span. J Soc Pers Relatsh. 2022;39(5):1482–507. 10.1177/02654075211059193.

[77] Balaj M, Henson CA, Aronsson A, Aravkin A, Beck K, Degail C, et al. Effects of education on adult mortality: a global systematic review and meta-analysis. Lancet Public Health. 2024;9(3):e155–65. 10.1016/S2468-2667(23)00306-7. 38278172 10.1016/S2468-2667(23)00306-7 PMC10901745

[78] Oshio T. Widening disparities in health between educational levels and their determinants in later life: evidence from a nine-year cohort study. BMC Public Health. 2018;18(1):278. 10.1186/s12889-018-5181-7. 29471834 10.1186/s12889-018-5181-7 PMC5824535

[79] Suulamo UK, Remes HM, Tarkiainen LH, Martikainen PT. Long-term trends in mortality by living arrangements and the role of socioeconomic factors, Finland 1991–2020. Eur J Public Health. 2025;35(5):814–20. 10.1093/eurpub/ckaf068. 40345138 10.1093/eurpub/ckaf068 PMC12529274

[80] Holt-Lunstad J. Social connection as a critical factor for mental and physical health: evidence, trends, challenges, and future implications. World Psychiatry. 2024;23(3):312–32. 10.1002/wps.21224. 39279411 10.1002/wps.21224 PMC11403199

