# Supplementary material for: Social connection before and after the COVID-19 pandemic: results of the Belgian Health Interview Survey
Source: BMC Public Health. 2026 Mar 18;26:1373. doi: 10.1186/s12889-026-26932-1 (PMC13113019; doi:10.1186/s12889-026-26932-1)
Supplement: Supplementary file 1 — Supplementary Material. [file 12889_2026_26932_MOESM1_ESM.docx]

**Supplementary Material 1**

**Table 1***Socio-demographic sample characteristics*

|  |  | 2018 | | 2023-2024 | | Total sample (2018 + 2023-2024) | |
| --- | --- | --- | --- | --- | --- | --- | --- |
|  |  | *N* | Unweighted % | *N* | Unweighted % | *N* | Unweighted % |
| **Sex** |  |  |  |  |  |  |  |
|  | Men | 3818 | 47.28 | 2339 | 47.48 | 6157 | 47.36 |
|  | Women | 4257 | 52.72 | 2587 | 52.52 | 6844 | 52.64 |
| **Education** |  |  |  |  |  |  |  |
|  | No tertiary education | 4639 | 57.45 | 2575 | 52.27 | 7214 | 55.49 |
|  | Tertiary education | 3436 | 42.55 | 2351 | 47.73 | 5787 | 44.51 |
| **Living situation** |  |  |  |  |  |  |  |
|  | Living alone | 1863 | 23.07 | 1132 | 22.98 | 2995 | 23.04 |
|  | Not living alone | 6212 | 76.93 | 3794 | 77.02 | 10006 | 76.96 |
| **Age group** |  |  |  |  |  |  |  |
|  | 15-24 years old | 728 | 9.02 | 374 | 7.59 | 1102 | 8.48 |
|  | 25-34 years old | 1055 | 13.07 | 635 | 12.89 | 1690 | 13.00 |
|  | 35-44 years old | 1308 | 16.20 | 748 | 15.18 | 2056 | 15.81 |
|  | 45-54 years old | 1485 | 18.39 | 741 | 15.04 | 2226 | 17.12 |
|  | 55-64 years old | 1468 | 18.18 | 916 | 18.60 | 2384 | 18.34 |
|  | 65-74 years old | 1140 | 14.12 | 828 | 16.81 | 1968 | 15.14 |
|  | 75+ years old | 891 | 11.03 | 684 | 13.89 | 1575 | 12.11 |

*Note.* The table shows sample sizes and unweighted percentages of the total sample (i.e., combining the waves of 2018 and 2023-2024).

**Supplementary Material 2**

**Survey questions on social connection**

*Frequency of social contacts*

How often do you usually have contact with relatives, children, friends, acquaintances,…?

- At least once per week
- At least once per month
- At least 3 to 4 times per year
- At least once per year
- Not at all

*Social support: OSSS-3 question 1*

How many people are so close to you that you can count on them if you have serious personal problems?

- None
- 1 or 2
- 3 to 5
- 6 or more

*Social support: OSSS-3 question 2*

How much interest and concern do people show in what you are doing?

- A lot of concern and interest
- Some concern and interest
- Uncertain
- Little concern and interest
- No concern and interest

*Social support: OSSS-3 question 3*

How easy is it to get practical help from neighbors if you should need it?

- Very easy
- Easy
- Possible
- Difficult
- Very difficult

*Social satisfaction*

How satisfying do you find your social contacts?

- Very satisfying
- Rather satisfying
- Rather unsatisfying
- Very unsatisfying

**Supplementary Material 3**

As the dichotomization of indicators in the main analyses has its limitations (see Discussion), we repeated the main analyses with non-dichotomized versions of the social connection indicators. This was done using cumulative logistic regression models that included the same predictors as the selected models in the main analyses. The results of these additional analyses are reported here.

**Social frequency**

For the non-dichotomized analysis of social frequency, we used the original social frequency question, which contained five response options (see SM 2). The results of the non-dichotomized analysis of social frequency were completely in line with the results of the main dichotomized analysis. This means that there was no main effect of year (*p* = .860). This means that the distribution of individual probabilities of each response option was similar in 2018 as compared to 2023-2024. Additionally, as was the case in the main analyses, there was a significant main effect of education (*F*(1, 7831) = 20.12, *p* < .001), household situation (*F*(1, 7831) = 5.83, *p* = .016) and sex (*F*(1, 7831) = 10.39, *p* = .001). The direction of these effects were completely in line with the main analyses. The distribution of individual response probabilities for education, household situation and sex can be found in Table 2.

**Table 2**

*Distribution of social frequency response probabilities for education, household situation and sex.*

|  |  | IP_weekly_ | IP_monthly_ | IP_3-4x per year_ | IP_once a year_ | IP_not at all_ |
| --- | --- | --- | --- | --- | --- | --- |
| **Education** |  |  |  |  |  |  |
|  | Non-tertiary | 0.850 | 0.096 | 0.037 | 0.008 | 0.008 |
|  | Tertiary | 0.902 | 0.064 | 0.024 | 0.005 | 0.005 |
| **Household situation** |  |  |  |  |  |  |
|  | Living alone | 0.846 | 0.098 | 0.038 | 0.009 | 0.008 |
|  | Not living alone | 0.882 | 0.077 | 0.029 | 0.007 | 0.006 |
| **Sex** |  |  |  |  |  |  |
|  | Men | 0.859 | 0.091 | 0.035 | 0.008 | 0.008 |
|  | Women | 0.887 | 0.074 | 0.028 | 0.006 | 0.006 |

*Note.* IP = individual probability, only shown for significant effects in the cumulative logistic regression model.

**Social support**

For the non-dichotomized analysis of social support, we used the categorical version of the social support indicators that distinguishes between low, moderate and high social support. This was done using same scoring guidelines of the OSSS-3, discussed in the Methods section of the main text. Raw scores and uncategorized sum scores were not used, in order to stay in line with the validated guidelines of the OSSS-3. The results of the non-dichotomized analysis of social support were largely in line with the results of the main dichotomized analysis. There was no main effect of year (*p* = .083). This means that the distribution of individual probabilities of each response option was similar in 2018 as compared to 2023-2024. In addition, as was the case in the main analyses, there was a significant main effect of education (*F*(1, 7768) = 67.34, *p* < .001), in the same direction of the main effect in the main analysis (i.e., more low support in those without tertiary education). The distribution of individual response probabilities for education can be found in Table 3. However, importantly, the main effect of household situation observed in the main dichotomized analyses, was not found in the non-dichotomized analyses (*p* = .174). This means that the distribution of the probabilities of each of the three social support categories was not different for those living alone versus those not living alone.

**Table 3**

*Distribution of social support category probabilities for education.*

|  |  | IP_low support_ | IP_moderate support_ | IP_high support_ |
| --- | --- | --- | --- | --- |
| **Education** |  |  |  |  |
|  | Non-tertiary | 0.188 | 0.519 | 0.293 |
|  | Tertiary | 0.124 | 0.472 | 0.404 |

*Note.* IP = individual probability, only shown for significant effects in the cumulative logistic regression model.

**Social satisfaction**

For the non-dichotomized analysis of social satisfaction, we used the original social satisfaction question, which contained four response options (see SM 2). The results of the non-dichotomized analysis of social satisfaction were not in line with the results of the main dichotomized analysis. The only result that was found in both analyses was the significant main effect of household situation (*F*(1, 7837) = 4.52, *p* = .034), showing lower probabilities for response options indicating social satisfaction in those living alone versus those not living alone (see Table 4). Importantly, there were some differences between the dichotomized and non-dichotomized analyses. First, whereas there was no significant main effect in the dichotomized analysis, the main effect of year was significant in the non-dichotomized analysis (*F*(1, 7837) = 5.54, *p* = .019). The overall prevalence of satisfaction (combining “very satisfied” and “rather satisfied) did not change (result of dichotomized analysis), but the distribution of response options within that collapsed category of “satisfied” did change. More specifically, the individual response probability of “very satisfied” was higher in 2018 than in 2023-2024, and the individual response probability of “rather satisfied” was lower in 2018 than in 2023-2024 (see Table 4) . This means that there was a shift within the “satisfied” category from “very satisfied” to “rather satisfied”, indicating a subtle change in the prevalence of social satisfaction from the pre-pandemic to the post-pandemic measurement. Second, whereas the dichotomized analysis showed a significant main effect of education, the non-dichotomized analysis no longer showed such a difference (*p* = .130). Third, the main effect of age, observed in the dichotomized analysis, was also not observed in the non-dichotomized analysis (*p* = .910). However, when using age group (i.e., 15-24, 25-64 and 65+) as a categorical predictor instead of age as a continuous predictor, there was a main effect of age group on social satisfaction (*F*(2, 7049) = 7.45, *p* = .001). Interestingly, this shows that the response probabilities of “very satisfied” are higher in 15-24 year-olds and those aged 65 or older, compared to those aged 25 to 64 (see Table 4).

**Table 4**

*Distribution of social satisfaction response probabilities for year, household situation and age group.*

|  |  | IP_very satisfied_ | IP_rather satisfied_ | IP_rather unsatisfied_ | IP_very unsatisfied_ |
| --- | --- | --- | --- | --- | --- |
| **Year** |  |  |  |  |  |
|  | 2018 | 0.337 | 0.578 | 0.068 | 0.016 |
|  | 2023-2024 | 0.301 | 0.601 | 0.079 | 0.019 |
| **Household situation** |  |  |  |  |  |
|  | Living alone | 0.304 | 0.600 | 0.078 | 0.019 |
|  | Not living alone | 0.329 | 0.583 | 0.071 | 0.017 |
| **Age group** |  |  |  |  |  |
|  | 15-24 | 0.344 | 0.573 | 0.066 | 0.017 |
|  | 25-64 | 0.299 | 0.604 | 0.080 | 0.021 |
|  | 65+ | 0.358 | 0.565 | 0.062 | 0.015 |

*Note.* IP = individual probability, only shown for significant effects in the cumulative logistic regression model.
